# Neurological disease caused by Oropouche virus in northern Brazil: should it be included in the scope of clinical neurological diseases?

**DOI:** 10.1007/s13365-021-00987-9

**Published:** 2021-06-11

**Authors:** Jannifer O. Chiang, Rafael S. Azevedo, Maria C. A. Justino, Haroldo J. Matos, Hideraldo L. S. Cabeça, Sandro P. Silva, Daniele F. Henriques, Eliana V. P. Silva, Gabriela S. S. Andrade, Pedro FC. Vasconcelos, Lívia C. Martins, Raimunda S. S. Azevedo

**Affiliations:** 1 Institutional Program for Scientific Initiation Scholarships (PIBIC), Evandro Chagas Institute. Ananindeua, Pará, Brazil; 2 Department of Virology, Evandro Chagas Institute. Ananindeua, Pará, Brazil; 3 Epidemiology Service, Evandro Chagas Institute, Ananindeua, Pará, Brazil; 4 Department of Arbovirology and Hemorrhagic Fevers, Evandro Chagas Institute, Ananindeua, Pará, Brazil; 5 Ophir Loyola Hospital. Belém, Pará, Brazil

**Keywords:** Oropouche virus, Arbovirus, Central nervous system, Neurological disease, Neglected disease

## Abstract

We describe two neurological cases of Oropouche virus infection in northern Brazil, where the virus is endemic but neglected as a pathogen. This study reiterates the necessity of developing protocols for diagnosing infections and training medical personnel to recognize the pathogenicity of Oropouche virus in neurological infections.

## Introduction

The emergence of encephalitic arboviruses in Brazil requires the attention of health and surveillance professionals to investigate neurological impairment as a possible symptom of arbovirus infections (Vieira et al. 2015, 2018b). Several arboviruses, including *West Nile virus* (WNV),* Zika virus* (ZIKV), *dengue virus* (DENV), *Saint Louis encephalitis virus* (SLEV), and *chikungunya virus* (CHIKV), can cause infection or impairment to the CNS (Vieira et al. 2018a; Carmo et al. 2019). Certain arboviruses, including *Oropouche virus* (OROV), are less known to be associated with CNS infections.

OROV is an arbovirus classified into the family *Peribunyaviridae*, genus *Orthobunyavirus* (order *Bunyavirales*), and is transmitted via the urban cycle to humans by *Culicoides paraensis*. The virus circulates in Central America (Panama and Trinidad and Tobago) and South America (Brazil and Peru), causing more than 30 epidemics and over half a million clinical cases of Oropouche fever. The disease caused by OROV is characterized by acute febrile illness, accompanied by headache, arthralgia, myalgia, photophobia, and other systemic manifestations. Some patients exhibit neurological manifestations, such as aseptic meningitis or even meningoencephalitis, but this is rare or underdetected. In some cases, the symptoms of Oropouche fever commonly reappear a few days after the end of the first febrile episode; however, the symptoms are usually less severe, and patients generally recover completely without sequelae, even in severe cases (Romero-Alvarez and Escobar 2018; Sakkas et al. 2018).

In the 1980s, during an investigation of Oropouche fever in Belém, Brazil, Pinheiro et al. reported cases of a neurological disease diagnosed as aseptic meningitis (Pinheiro et al. 1981). Later, de Bastos et al. detected by RT-PCR OROV from the cerebrospinal fluid (CSF) of three patients in Amazonas, Brazil, during 2006 and 2007 (de Bastos et al. 2012). The clinical symptoms associated with OROV neurological cases are rare or under detected owing to the lack of knowledge and specific diagnostic methods. Therefore, we aimed to describe two human clinical cases with neurological symptoms associated with OROV infection.

## Case descriptions

**Case 1.** A 73-year-old woman living in Belém City, Brazil, reported a fall from standing height due to sudden dizziness. While in medical care, she was conscious, time and space-oriented, and reported headache, cramps, and an isolated episode of generalized tonic–clonic seizure. A blood count confirmed leukocytopenia, lymphocytosis, and thrombocytopenia (leukocytes 3,244 cells/µL; lymphocytes 14,300 cells/µL; platelets 104,800 cells/µL). Computed tomography (CT) of the head and magnetic resonance imaging of the brain showed no alterations; however, the electroencephalogram identified episodes of slow waves in the temporal lobe. The patient was discharged from the hospital 48 h after admission, without convulsions and in a generally good condition. The next day, she experienced vomiting, diarrhea, fever (38 °C), and a new convulsive episode. She was clinically re-evaluated at the hospital, and while normotensive, a sample of clear CSF was collected 4 days after the onset of symptoms that tested negative for bacterial and fungal growth.

**Fig. 1:**
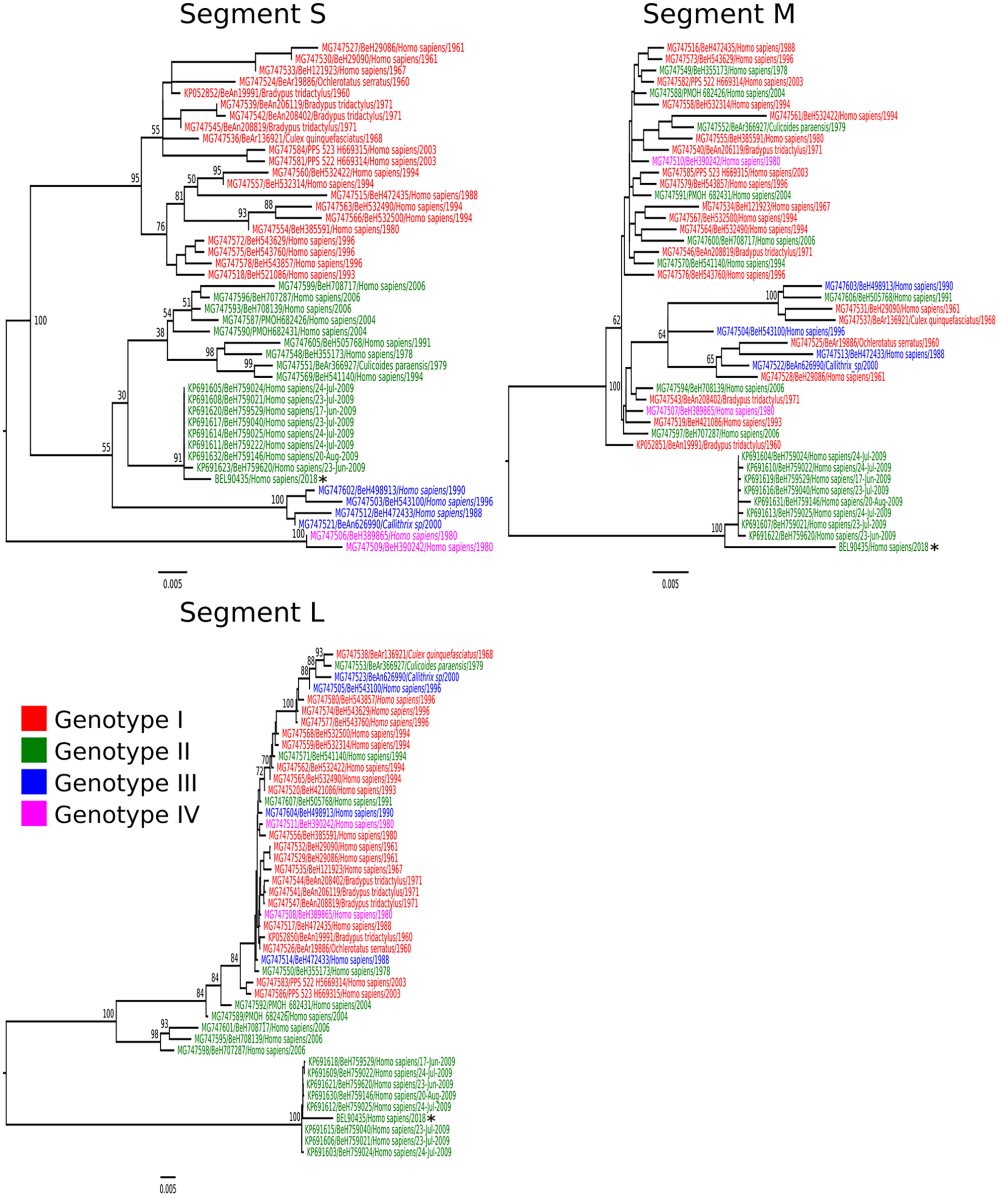
Phylogenetic tree of the different Oropouche virus genotypes generated using maximum likelihood methodology. The phylogenetic tree was created based on nucleotide sequences of small (S), medium (M), and large (L) RNA segments. The scale bar indicates the evolutionary distance in numbers of nucleotides substitutions per site, and the principal bootstrap support levels are indicated. The tips of tree are colored according to genotype. Star indicates sample obtained from case 1 (BEL90435). GenBank accession numbers MT879228, MT879229, and MT879230: RNA segments S, L, and M, respectively

In the Brazilian National Reference Laboratory for arboviruses, quantitative reverse-transcription polymerase chain reaction (RT-qPCR) for CHIKV (Lanciotti et al. 2007), DENV (Santiago et al. 2013), SLEV (Lanciotti and Kerst 2001), WNV (Lanciotti et al. 2000), and ZIKV (Lanciotti et al. 2008) using CSF was negative; however, OROV was detected (Naveca et al. 2017). OROV was isolated in Vero cell culture from the CSF sample (Gubler et al. 1984) and was identified as genotype II by nucleotide sequencing (Fig. 1). On the 10th day after disease onset, a serum sample was obtained and specific anti-OROV immunoglobulin M (IgM) and total antibodies against OROV were detected using IgM enzyme-linked immunosorbent assay (ELISA) and hemagglutination inhibition (HI) tests, respectively. Serological test results were negative for CHIKV (Martin et al. 2000), DENV (Henriques et al. 2020), SLEV (Henriques et al. 2020), WNV (Henriques et al. 2020), and ZIKV (Henriques et al. 2020). We concluded that the patient suffered a recent OROV infection characterized by encephalitis. She was discharged, after which she recovered without further symptoms.

**Case 2.** A 52-year-old man living in Ananindeua City, Brazil, was clinically examined on the third day of illness and experienced high fever, with episodes of focal seizures persisting for 3 days. Clinical examination revealed altered gait, muscle weakness, and absence of signs of meningeal irritation, but a positive Romberg sign; the CT scans and hemogram results were normal. During examination in the National Reference Laboratory, RT-qPCR using serum tested negative for the RNA of CHIKV (Lanciotti et al. 2007), DENV (Santiago et al. 2013), SLEV (Lanciotti and Kerst 2001), WNV (Lanciotti et al. 2000), and ZIKV (Lanciotti et al. 2008), but positive for OROV (Naveca et al. 2017). Similarly, IgM-ELISA was negative for CHIKV, DENV (Henriques et al. 2020), SLEV (Henriques et al. 2020), WNV (Henriques et al. 2020), and ZIKV (Henriques et al. 2020), but positive for OROV-IgM. On the 11th day after onset of symptoms, CSF was collected and IgM-ELISA confirmed OROV infection. Moreover, HI test (Clarke and Casals 1958) showed the presence of total antibodies against OROV in both serum and CSF samples.

It is noteworthy that approximately 30 days before the clinical symptoms mentioned previously, the patient developed another clinical condition of high fever accompanied by headache, myalgia, and arthralgia that persisted for 2 weeks without a clear etiological diagnosis at the time. The patient developed insomnia, right hearing loss, and horizontal nystagmus and thus sought for specialized investigation at the National Reference Laboratory for Arboviruses, as described above. It is important to clarify that sample collection for the second clinical investigation was considered after the appearance of neurological symptoms. The case was diagnosed as OROV-associated encephalitis and the patient recovered without further sequelae.

## Discussion

Neurological symptoms associated with OROV infections are rarely observed in humans (Pinheiro et al. 1982; Vernal et al. 2019), and the mechanisms used by the virus to reach the brain are unknown, although experimental infections using animal models (mouse and hamsters) confirm OROV tropism in brain cells (Rodrigues et al. 2011; Santos et al. 2012, 2014). Here, we contribute with the description and laboratory confirmation of two cases of human neurological infections associated with OROV.

OROV has been detected only in a few countries in south and central America (Trinidad and Tobago, Panama, Peru, and Brazil), but the majority of OROV human infections are reported in Brazil, with a cumulative estimate of over 450 million infections since the first OROV epidemic in 1961 (Romero-Alvarez and Escobar 2018). However, this number is probably underestimated, since accurate clinical investigation and accessible diagnostic tests are still lacking in many countries; in Brazil, a few laboratories have the capacity to detect OROV infection, which may reflect the low numbers of OROV neurological cases.

Meningitis cases associated with OROV infection were first observed and described by Pinheiro and collaborators in 1980, during an Oropouche fever outbreak in three municipalities (Belém, Vigia, and Curuçá) in the state of Pará, Brazil (Pinheiro et al. 1982). The neurological cases identified until now occurred only in northern Brazil (Pará and Amazonas states), where the virus is endemic and periodically causes small outbreaks and/or isolated cases of febrile disease (de Bastos et al. 2012), and there are laboratories with specific tests for OROV diagnosis. Outside of this region, an OROV case was reported in southeastern Brazil; however, the patient had traveled to a municipality of the metropolitan region of Belém, Brazil (Vernal et al. 2019).

There are no clear clinical patterns regarding the neurological symptoms caused by OROV infection; however, in general, the most commonly reported symptoms include high fever, intense and severe headache, balance dysfunction, and photophobia. Further, nausea, vomiting, stiff neck, moderate mental confusion, and lethargy are reported in few cases. Seizures have not been reported thus far, which were observed in both the cases in this study.

The identification of neurological symptoms associated with OROV infection until now is a result of retrospective analysis, using CSF collected from neurological cases in Pará and Amazonas states, or by chance when patients were examined by professionals with knowledge on the pathogenicity of OROV to cause neurological disease. Case 1 is an appropriate example since the patient was examined by experienced professionals with knowledge of arbovirus infections, and a broad range of laboratory parameters were investigated, resulting in OROV isolation from the CSF followed by molecular characterization, which is the gold standard of diagnostic methods for neurological cases.

Case 2 may represent a case of recurrence of symptoms, as previously described by Pinheiro et al., during OROV epidemics (Pinheiro et al. 1982). The patient was evaluated by a neurologist with expertise in arboviruses; the occurrence of symptoms 30 days before seeking medical assistance and symptoms characteristic of Oropouche fever, which lasted for 2 weeks, were suggestive of OROV infection. However, after an apparent improvement of the symptoms, the patient developed another clinical condition with neurological symptoms as observed before (Pinheiro et al. 1982).

There are four different genotypes of OROV (I, II, III, and IV) currently circulating in the south and central Americas (Travassos da Rosa et al. 2017). Neurological cases are associated with OROV genotypes I and II; however, experimental studies investigating the differences in neurological symptoms caused by the four OROV genotypes are necessary to understand the physiopathology of the disease.

These two cases stress the necessity of training health workers, especially medical staff, to recognize the pathogenicity of OROV in neurological infections and amplifying the diagnostic capacity for OROV. Additionally, OROV should be recognized as a potential pathogen in cases of fever associated with neurological symptoms, regardless of meningitis and meningoencephalitis diagnosis, especially in patients with a history of exposure to arboviruses.

The poor epidemiological and pathological OROV scenario, regarding knowledge of the scientific community, especially in northern Brazil where the virus is endemic, reflects how OROV is neglected by the health professionals working in this region. Indeed, OROV is usually not considered in the investigation of etiological agents of neurological infections, including meningitis and encephalitis, indicating that studies are needed to improve the understanding of neurological OROV infections and amplify the laboratorial capacity for OROV diagnosis. We believe that such studies may lead to the eventual inclusion of OROV infections within the scope of clinical neurological diseases and in the improvement of epidemiological data of OROV in Brazil.

## References

[CR1] Carmo RL, do, Alves Simão AK, Amaral LLF do,  (2019). Neuroimaging of emergent and reemergent infections. Radiographics.

[CR2] Clarke DH, Casals J (1958). Techniques for hemagglutination and hemagglutination-inhibition with arthropod-borne viruses. Am J Trop Med Hyg.

[CR3] de Bastos M, S, Figueiredo LTM, Naveca FG,  (2012). Identification of Oropouche Orthobunyavirus in the cerebrospinal fluid of three patients in the Amazonas, Brazil. Am J Trop Med Hyg.

[CR4] Gubler DJ, Kuno G, Sather GE (1984). Mosquito cell cultures and specific monoclonal antibodies in surveillance for dengue viruses. Am J Trop Med Hyg.

[CR5] Henriques DF, Nunes JAL, Anjos MV (2020). Evaluation of immunoglobulin M-specific capture enzyme-linked immunosorbent assays and commercial tests for flaviviruses diagnosis by a national reference laboratory. J Virol Methods.

[CR6] Lanciotti RS, Kerst AJ (2001). Nucleic acid sequence-based amplification assays for rapid detection of West Nile and St. Louis encephalitis viruses. J Clin Microbiol.

[CR7] Lanciotti RS, Kerst AJ, Nasci RS (2000). Rapid detection of West Nile Virus from Human clinical specimens, field-collected mosquitoes, and avian samples by a TaqMan reverse transcriptase-PCR assay. J Clin Microbiol.

[CR8] Lanciotti RS, Kosoy OL, Laven JJ (2007). Chikungunya virus in US travelers returning from India, 2006. Emerg Infect Dis.

[CR9] Lanciotti RS, Kosoy OL, Laven JJ (2008). Genetic and serologic properties of Zika virus associated with an epidemic, Yap State, Micronesia, 2007. Emerg Infect Dis.

[CR10] Martin DA, Muth DA, Brown T (2000). Standardization of immunoglobulin M capture enzyme-linked immunosorbent assays for routine diagnosis of arboviral infections. J Clin Microbiol.

[CR11] Naveca FG, Nascimento VA, do, Souza VC de,  (2017). Multiplexed reverse transcription real-time polymerase chain reaction for simultaneous detection of Mayaro, Oropouche, and Oropouche-like viruses. Mem Inst Oswaldo Cruz.

[CR12] Pinheiro FP, Rocha AG, Freitas RB, Ohana BA, da Rosa T, Amélia PA, Rogério JS, Linhares AC (1982). Meningite associada às infecções por Vírus Oropouche. Revista Do Instituto De Medicina Tropical.

[CR13] Pinheiro FP, Travassos da Rosa AP, Travassos da Rosa JF (1981). Oropouche virus. I. A review of clinical, epidemiological, and ecological findings. Am J Trop Med Hyg.

[CR14] Rodrigues AH, Santos RI, Arisi GM (2011). Oropouche virus experimental infection in the golden hamster (Mesocrisetus auratus). Virus Res.

[CR15] Romero-Alvarez D, Escobar LE (2018). Oropouche fever, an emergent disease from the Americas. Microbes Infect.

[CR16] Sakkas H, Bozidis P, Franks A, Papadopoulou C (2018). Oropouche Fever: A Review Viruses.

[CR17] Santiago GA, Vergne E, Quiles Y (2013). Analytical and clinical performance of the CDC real time RT-PCR assay for detection and typing of dengue virus. PLoS Negl Trop Dis.

[CR18] Santos RI, Almeida MFP, Paula FE (2012). Experimental infection of suckling mice by subcutaneous inoculation with Oropouche virus. Virus Res.

[CR19] Santos RI, Bueno-Júnior LS, Ruggiero RN (2014). Spread of oropouche virus into the central nervous system in mouse. Viruses.

[CR20] Travasos da Rosa JF, de Souza WM, de Paula PF (2017). Oropouche virus: clinical, epidemiological, and molecular aspects of a neglected orthobunyavirus. Am J Trop Med Hyg.

[CR21] Vernal S, Martini CCR, da Fonseca BAL (2019). Oropouche virus-associated aseptic meningoencephalitis, southeastern Brazil. Emerg Infect Dis.

[CR22] da Vieira MA, C e S, Costa CHN, Linhares A da C,  (2018). Potential role of dengue virus, chikungunya virus and Zika virus in neurological diseases. Mem Inst Oswaldo Cruz.

[CR23] Vieira MACS, Castro AAS, Henriques DF et al (2018b) Encephalitis associated with Zika virus infection and reactivation of the varicella-zoster virus in a Brazilian child Rev Soc Bras Med Trop 51 10.1590/0037-8682-0447-2017 10.1590/0037-8682-0447-2017 29972576

[CR24] Vieira MACS, Romano APM, Borba AS (2015). West Nile virus encephalitis: the first human case recorded in Brazil. Am J Trop Med Hyg.

